# Value of CRP, albumin, and lymphocyte index in predicting survival of patients with gastrointestinal malignancies: a systematic review and meta-analysis

**DOI:** 10.3389/fonc.2025.1592794

**Published:** 2025-07-16

**Authors:** Hui Li, Zhaozhao Mo, Guojun Tong

**Affiliations:** Department of Anorectal Surgery, Huzhou Central Hospital, Affiliated Central Hospital of HuZhou University, Huzhou, Zhejiang, China

**Keywords:** inflammation, biomarker, cancer, survival, prognosis

## Abstract

**Background:**

We conducted this systematic review to present high-quality evidence on the prognostic ability of CRP, albumin, and lymphocyte (CALLY) index for gastrointestinal (GI) malignancies.

**Methods:**

PubMed, Embase, Scopus, Web of Science, and Wanfang databases were searched till 15^th^ January 2025 for studies reporting the prognostic ability of CALLY for all GI malignancies. Hazard ratios (HR) were pooled in a random-effect model for overall survival (OS) and progression-free survival (PFS).

**Results:**

18 studies were included. CALLY index was found to be a significant predictor of poor OS (HR: 1.89 95% CI: 1.720, 2.077 I^2^ = 12%) and PFS (HR: 1.617 95% CI: 1.444, 1.809 I^2^ = 1%) in GI malignancies. Low CALLY was a significant predictor of OS in pancreatic cancer (HR: 1.772 95% CI: 1.279, 2.456), cholangiocarcinoma (HR: 2.07 95% CI: 1.106, 3.875), colorectal liver metastasis (HR: 1.67 95% CI: 1.032, 2.702), gastric cancer (HR: 1.884 95% CI: 1.606, 2.210 I^2^ = 15%), colorectal cancer (HR: 2.284 95% CI: 1.737, 3.004 I^2^ = 0%), hepatocellular cancer (HR: 1.649 95% CI: 1.308, 2.079 I^2^ = 0%), and esophageal cancer (HR: 2.133 95% CI: 1.607, 2.831 I^2^ = 62%). Likewise, low CALLY was associated with worse PFS in pancreatic cancer (HR: 1.289 95% CI: 1.006, 1.652), esophageal cancer (HR: 2.171 95% CI: 1.543, 3.056 I^2^ = 0%), hepatocellular cancer (HR: 1.468 95% CI: 1.195, 1.801 I^2^ = 0%), gastric cancer (HR: 1.904 95% CI: 1.539, 2.356 I^2^ = 0%) and cholangiocarcinoma (HR: 2.13 95% CI: 1.163, 3.902). Random-effect meta-regression using sample size, age, male gender, TNM stage III/IV, lymph node metastasis, CALLY cut-off, low CALLY percentage, and follow-up as moderators were non-significant.

**Conclusions:**

CALLY can be a simple and easy-to-use prognostic marker for GI malignancies. Further research is needed to decipher its role in specific GI malignancies and improve the quality of evidence.

**Systematic review registration:**

https://www.crd.york.ac.uk/prospero/, identifier CRD42025636999.

## Introduction

Globally, cancer remains a leading cause of early death representing a significant barrier to additional increases in life expectancy in the coming decades (1). Worldwide mortality data shows that malignancy is among the top four non-communicable diseases causing about 10 million deaths in 2020 (2). About 1/4^th^ of all cancers and approximately 1.3^rd^ of all cancer-attributable deaths are due to gastrointestinal (GI) cancers (3). There seems to be an uneven distribution of GI cancers with hepatocellular carcinoma (HCC), esophageal, and gastric, cancer being common in Asia while pancreatic and colorectal (CRC) being more common in Western populations (3). Nonetheless, the poor prognosis associated with most GI malignancies remains a cause of concern and has been the focus of medical research (4). Availability of robust biomarkers can help in understanding the prognosis of cancer patients allowing rationalization of therapy, prioritization of those at high risk, and also aid in patient counselling.

There has been a surge in inflammatory, immune, and malnutrition markers for assessing cancer prognosis in recent times. Numerous indices like neutrophil-lymphocyte ratio, lymphocyte-monocyte ratio, platelet-lymphocyte ratio, C-reactive protein (CRP), Glasgow Prognostic Score, systemic immune-inflammation index (SII), and systemic immune response index (SIRI) have been explored for in oncology but questions persist on the most optimal marker (5, 6). A combination marker namely, the CRP-albumin-lymphocyte (CALLY) index has been recently suggested. It combines the inflammatory status (CRP), immune function (lymphocytes), and nutritional status (albumin) of an individual in a single index thereby overcoming the limitations of previously mentioned markers. Since chronic inflammation, poor immune function, and malnutrition have all been linked with worse outcomes in cancer patients (7), the combined CALLY index seems to be an attractive prognostic indicator in oncology. Studies have demonstrated that CALLY can predict outcomes in head and neck cancer (8), lung cancer (9), gastric cancer (10), CRC (11), and several other malignancies (7). However, there seems to be no high-quality evidence in the literature assessing its prognostic ability in GI cancer patients. We hereby conducted the first systematic review and meta-analysis examining if CALLY can predict outcomes in GI malignancies.

## Methods

We conducted this systematic review and meta-analysis in accordance with PRISMA guidelines (12) and began with protocol submission on PROSPERO (CRD42025636999). All relevant literature available on the websites of PubMed, Embase, Scopus, Web of Science, and Wanfang databases were searched a mix of free-text and MeSH/Emtree terms (**Supplementary Table 1**). We completed the search on 15^th^ January 2025. No restrictions were placed on location, publication time, or language. Two reviewers completed the search using the above-mentioned combination on all databases. The database search was also supplemented by hand-search of the reference list of included articles. Google Scholar was searched for gray literature.

All articles obtained from the databases were screened for relevancy to the review by two authors by abstract reading. All pertinent articles were further evaluated with full-text reading completed by both authors. If there were disagreements, these were solved by discussion and consensus.

We aimed to include all peer-reviewed studies, irrespective of the study design, conducted on adult patients with any type of GI malignancies. Studies were to assess the prognostic ability of pre-treatment CALLY by dividing the sample into high and low CALLY groups and reported multiple covariate-adjusted outcomes as effect size with 95% confidence intervals (CI). Outcomes of interest were overall survival (OS) and progression-free survival (PFS). We did not include conference abstracts, reviews, or case reports. We also excluded studies not reporting data for the meta-analysis. Particular care was taken to avoid studies with overlapping data to include articles with a maximum sample size from a particular database.

The authors first conducted a preliminary screening of all studies to check the type of baseline data. We then prepared a table to extract all relevant information which included: first author, year of publication, design, type of malignancy studied, sample size, age, gender, stage III/IV cancer, presence of lymph node metastasis, tumor size, treatment undertaken, CALLY cut-off, technique to determine the cut-off, prevalence of low CALLY scores in the sample, follow-up and the effect size of OS and PFS. If complete data was not reported, data imputation was not to be conducted and the study was to be excluded from the meta-analysis. No correspondence was carried out with any study authors.

We also examined the quality of the sourced literature by using the Newcastle-Ottawa Scale (13) which is commonly used for risk assessment of cohort studies. The Newcastle-Ottawa Scale determines bias in the article for selection of cohort, comparability of groups, and outcome assessment. Two authors judged each study for these domains and gave them scores ranging from zero to nine, the latter indicating high quality.

Data synthesis was done using Comprehensive Meta-analysis software (Version 3). The outcome data obtained from the studies was pooled to calculate hazard ratio (HR) and 95% CI in a random-effects model. This model was chosen as studies were conducted on different malignancies with different stages and treatments and therefore unlikely to have no heterogeneity. Nevertheless, subgroup analysis was conducted to assess the impact of CALLY on different GI malignancies. Heterogeneity among studies was assessed through the *I^2^* index. *I^2^* of over 50% and/or *P* < 0.05 indicated a large degree of heterogeneity.

We further conducted subgroup analyses based on location for OS. Random-effect meta-regression was also performed for important covariates namely, sample size, age, male gender, TNM stage III/IV, lymph node metastasis, CALLY cut-off, low CALLY percentage, and follow-up. Leave-on-out analysis function of the software was used to assess the impact of each study on the pooled analysis. Publication bias was tested by examining the symmetry of the funnel plot and Egger’s test.

## Results

**Figure 1:**
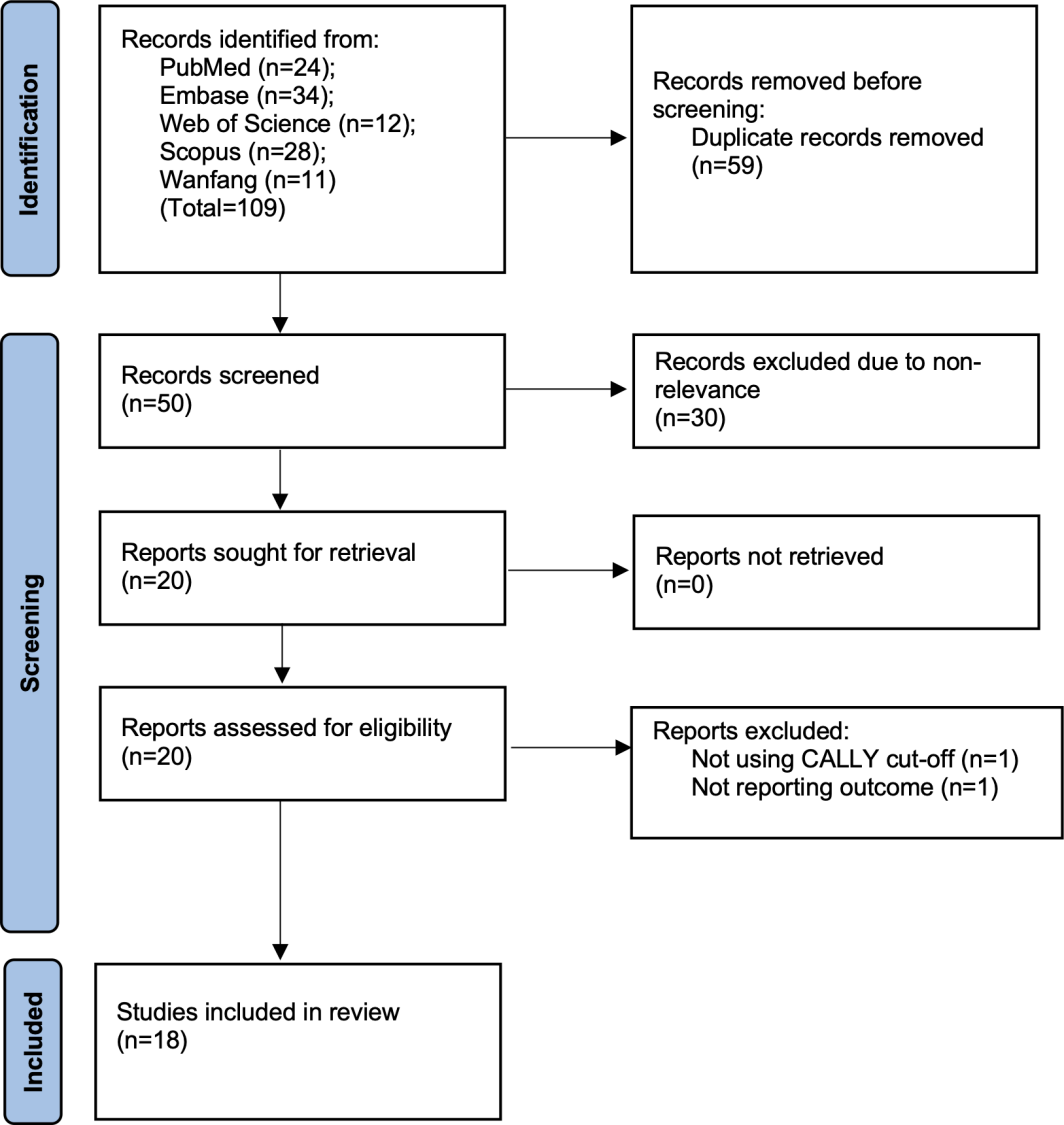
Search results and selection of study flowchart.

The literature search led to 109 hits on all databases. Removing duplicates and non-relevant studies, 20 studies were selected for further eligibility. Two were excluded as one did not use CALLY cut-off and did not report OS while another reported only complication rates. The remaining 18 studies were found to be eligible for inclusion (10, 11, 14–29) (**Figure 1**). There was no case of missing data which required clarification from the corresponding authors.

The included studies were conducted in four countries only with most data reported from Japan (**Table 1**). Only one study from Germany was based on the Western population. The studies were published over a time span of five years (2021–25) and all of them were in English language. Only one study was prospective while all others were retrospective. Maximum studies were available for gastric cancer (eight) followed by HCC and esophageal (three each). Two studies were on CRC, and one each on pancreas, cholangiocarcinoma, and colorectal liver metastasis (CLM). In total 8270 patients were included in the studies. All studies had predominantly elderly patients with male predominance. All studies estimated the CALLY index using baseline investigations performed before treatment. Limited data was available on specific clinicopathological characteristics of the malignancy. Surgery was the primary mode of treatment in most studies except for two where either transarterial chemoembolization (TACE) was used for treating HCC or either surgery, chemotherapy, or radiotherapy was used for esophageal cancer. Most studies used the receiver operating characteristic curve to assess the most suitable cut-off of CALLY. However, the cut-off varied from 1 to 6.96. The percentage of low CALLY patients varied from 17.8% to 87%. Thirteen studies reported 5-year follow-up data. In five studies, follow-up was <5 years. The majority of studies scored an eight on Newcastle-Ottawa Scale indicating good quality. One study scored a seven.

**Table 1:** Characteristics of studies included in the meta-analysis assessing the association between CALLY index and outcomes of GI malignancies.

Study ID	Design	Location	Type of malignancy	Sample size	Age (years)	Males (%)	TNM Stage (III/IV) (%)	Lymph node metastasis (%)	Tumor size (cm)	Treatment	CALLY cut-off	% with low CALLY	Method to determine cutoff	Follow-up (months)	NOS score
Lida 2021	RC	Japan	HCC	384	69.6	77.1	8.9	NR	4.4	Surgery	5	48	ROC	60	8
	RC	Japan	HCC	267	68.3	75.7	18.4	NR	4.6	Surgery	5	39.3	ROC	60	8
Muller 2021	RC	Germany	HCC	280	69.5	83.6	NR	NR	4.2	TACE	1	71.1	Optimal stratification	48	8
Tsunematsu 2022	RC	Japan	Cholangio-carcinoma	143	68	66	25	NR	NR	Surgery	3.5	87	ROC	42	8
Furukawa 2023 (17)	RC	Japan	CLM	183	65.5	69.3	NR	63.9	2.1	Surgery	4	55	NR	44.4	8
Yang 2023 (18)	RC	China	CRC	1260	60	60.9	73.8	NR	NR	NR	1.47	29.8	ROC	48	8
Zhang 2023 (19)	RC	China	Gastric	684	59	70	71	NR	NR	Surgery + chemotherapy	1.12	NR	ROC	60	8
	RC	China	Gastric	290	61	72	66	NR	NR	Surgery + chemotherapy	1.12	NR	ROC	60	8
Akdogan 2024	RC	Turkey	Gastric	74	60	62.2	62.2	63.5	NR	Surgery + chemotherapy	1.34	39.2	ROC	33.5	7
Aoyama 2024 (20)	RC	Japan	Esophagus	180	69	86.1	NR	67.8	NR	Surgery	5	48.9	NR	60	8
Aoyama 2024 (21)	RC	Japan	Gastric	259	70	NR	NR	NR	NR	Surgery	5	31.7	ROC	60	8
Fukushima 2024 (22)	RC	Japan	Gastric	826	68	71.8	17.6	32.8	NR	Surgery + chemotherapy	2	17.8	ROC	70.8	8
Hashimoto 2024 (23)	PC	Japan	Gastric	459	NR	34.6	NR	30.3	NR	Surgery	3.28	21.1	ROC	60	8
Kawahara 2024 (24)	RC	Japan	Pancreas	461	71	52.7	23.6	NR	3	Surgery	1.9	31	ROC	60	8
Ma 2024 (25)	RC	Japan	Esophagus	146	69	84.3	30.8	50.7	NR	Surgery	2.4	38.3	ROC	60	8
Nakashima 2024[ (26)	RC	Japan	Gastric	175	70	68	2	44	NR	Surgery	6.96	49	ROC	60	8
Okugawa 2024 (27)	RC	Japan	Gastric	486	NR	62.5	NR	34.2	NR	Surgery	4.93	NR	ROC	38.2	8
Sakurai 2024 (28)	RC	Japan	Gastric	617	70	55.6	21.4	37.4	NR	Surgery	1.19	47.1	ROC	60	8
Takeda 2024 (10)	RC	Japan	CRC	578	69	60	88.2	67	NR	Surgery	2	30	ROC	60	8
Jia 2025 (29)	RC	China	Esophagus	518	69	89.2	66.6	NR	NR	Surgery, Chemotherapy or radiotherapy	2.51	71.6	Maximum rank statistics	60	8

HCC, hepatocellular carcinoma; CRC, colorectal cancer; CLM, colorectal liver metastasis; ROC, receiver operating characteristics; RC, retrospective cohort; PC, prospective cohort; CALLY, C-reactive protein-albumin-lymphocyte.

**Figure 2:**
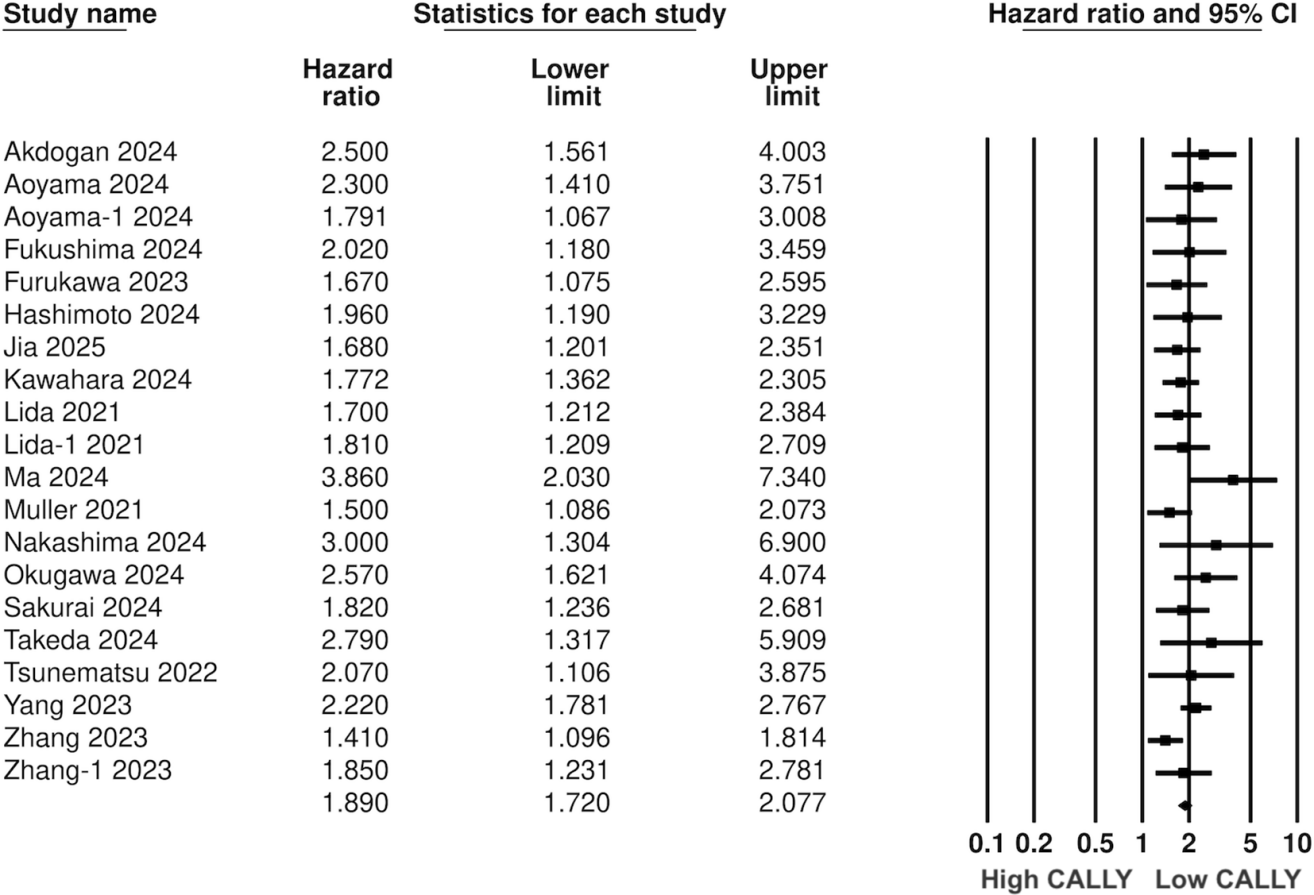
Composite meta-analysis for the association between CALLY and OS after GI malignancies.

**Figure 3:**
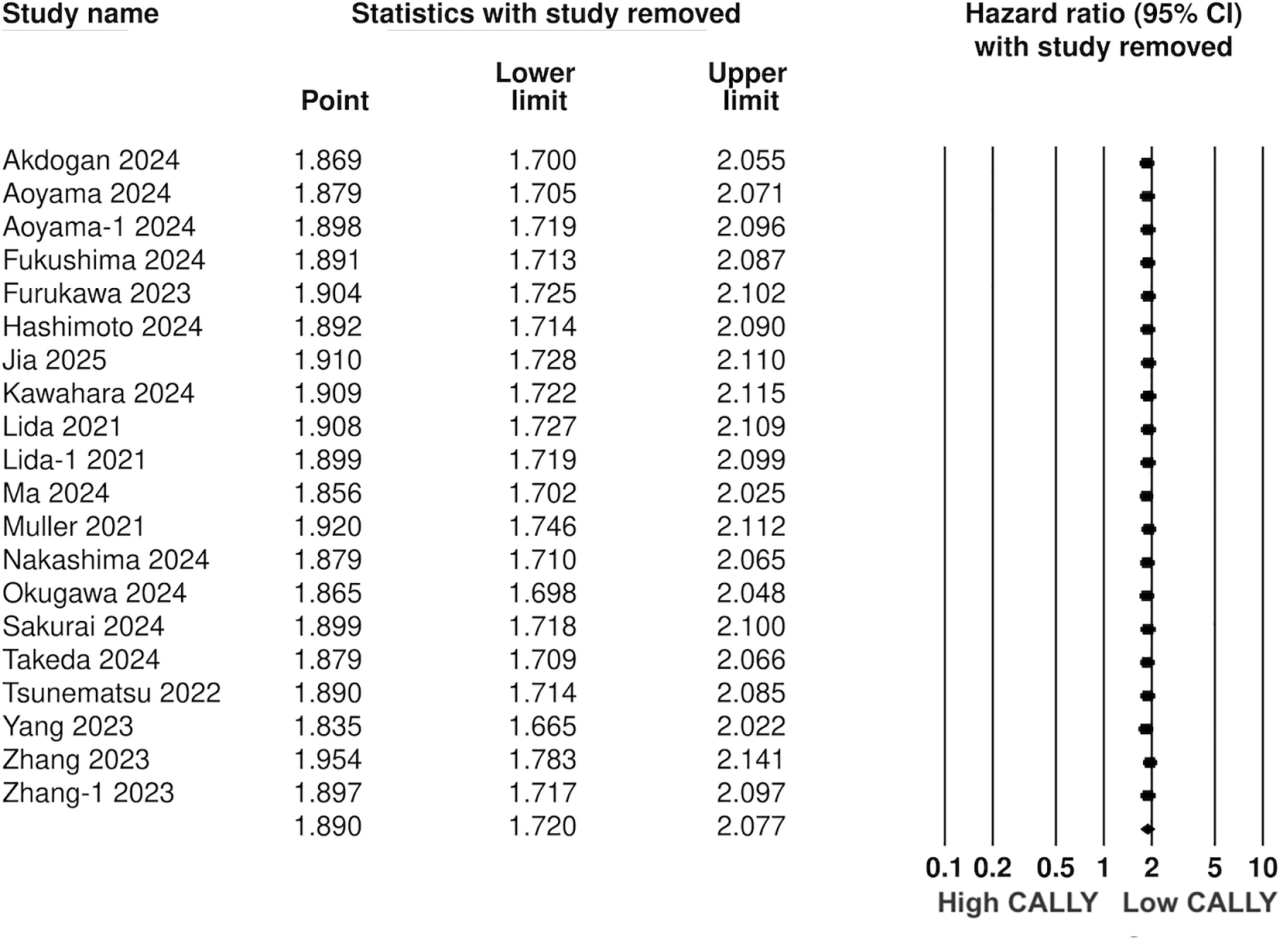
Sensitivity analysis for OS.

**Figure 4:**
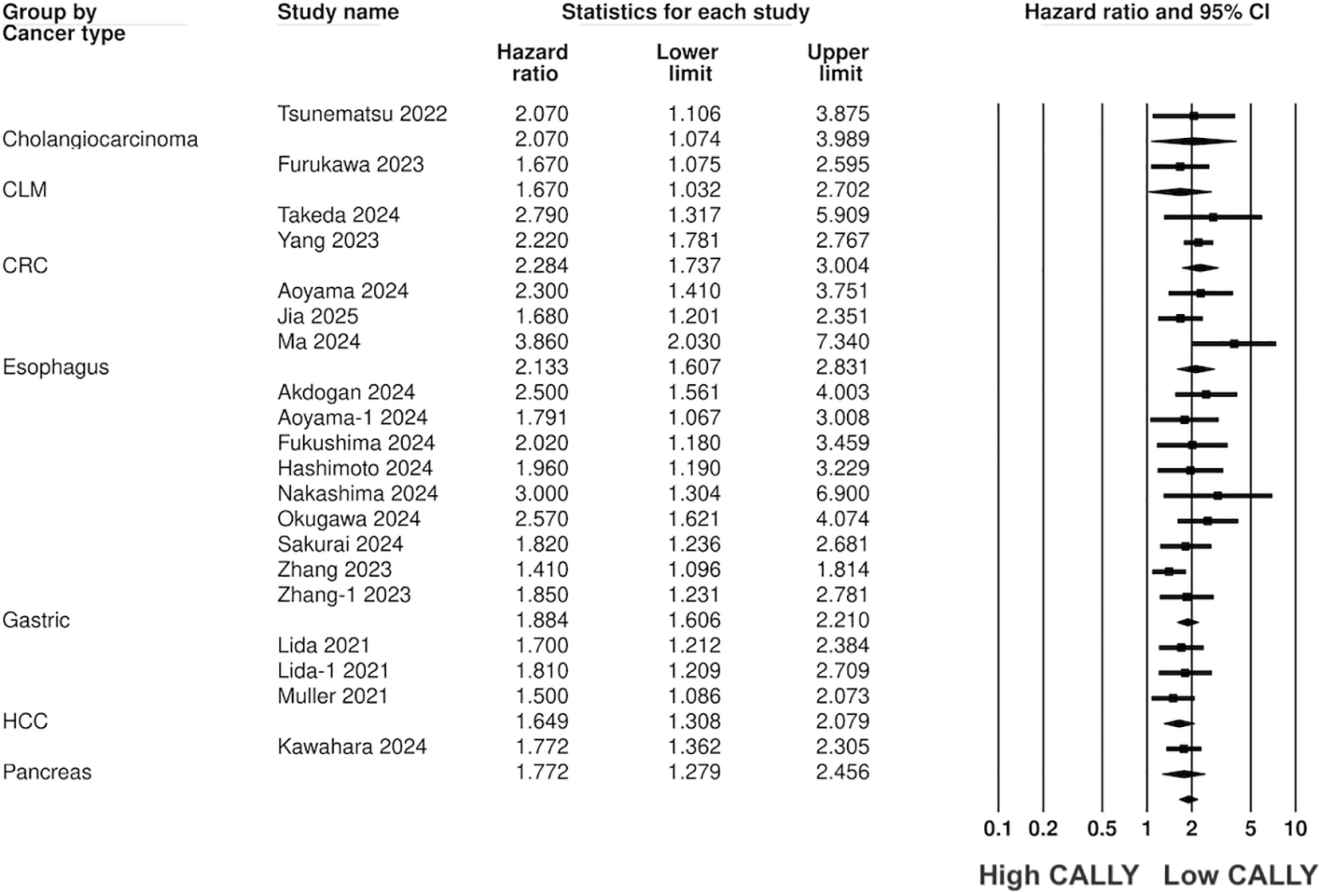
Subgroup analysis for OS based on different GI malignancies.

Pooled analysis of all included studies showed that low CALLY was a significant predictor of poor OS in GI malignancies (HR: 1.89 95% CI: 1.720, 2.077 I^2^ = 12%) (**Figure 2**). Removing one study at a time did not change the significance of the results (**Figure 3**). Publication bias was not noted on the funnel plot (**Supplementary Figure 1**). Egger’s test was not significant (p=0.69). Subgroup analysis of OS based on different malignancies is presented in **Figure 4**. Low CALLY was a significant predictor of OS in the singular studies on pancreatic cancer (HR: 1.772 95% CI: 1.279, 2.456), cholangiocarcinoma (HR: 2.07 95% CI: 1.106, 3.875), and CLM (HR: 1.67 95% CI: 1.032, 2.702). Similarly, results were found significant for gastric (HR: 1.884 95% CI: 1.606, 2.210 I^2^ = 15%), CRC (HR: 2.284 95% CI: 1.737, 3.004 I^2^ = 0%), HCC (HR: 1.649 95% CI: 1.308, 2.079 I^2^ = 0%), and esophageal cancer (HR: 2.133 95% CI: 1.607, 2.831 I^2^ = 62%) as well. On subgroup analysis based on location, we noted that results were significant for Japanese (HR: 1.981 95% CI: 1.735, 2.260 I^2^ = 0%) and Chinese studies (HR: 1.788 95% CI: 1.519, 2.104 I^2^ = 59%).

**Figure 5:**
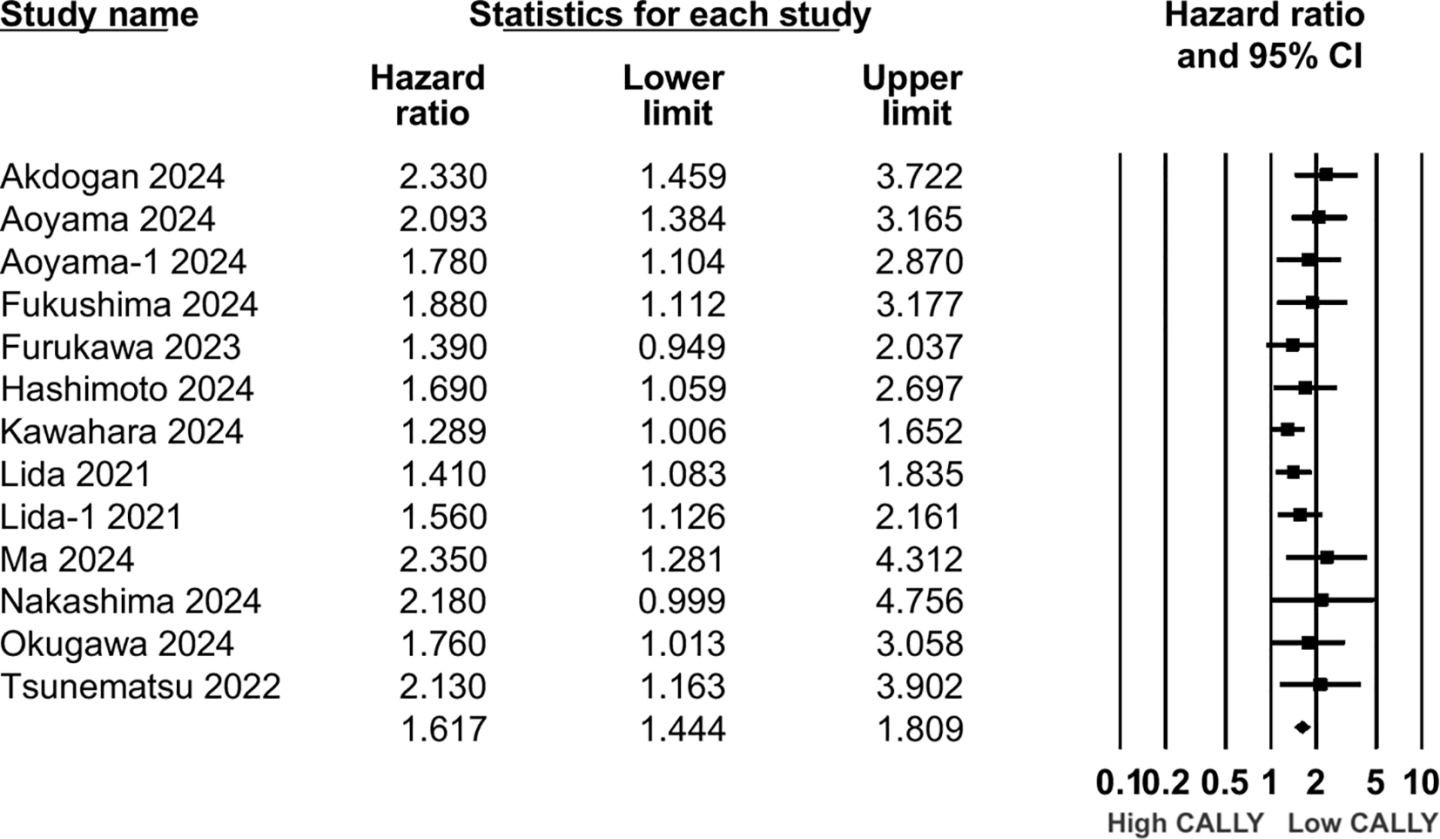
Composite meta-analysis for the association between CALLY and PFS after GI malignancies.

**Figure 6:**
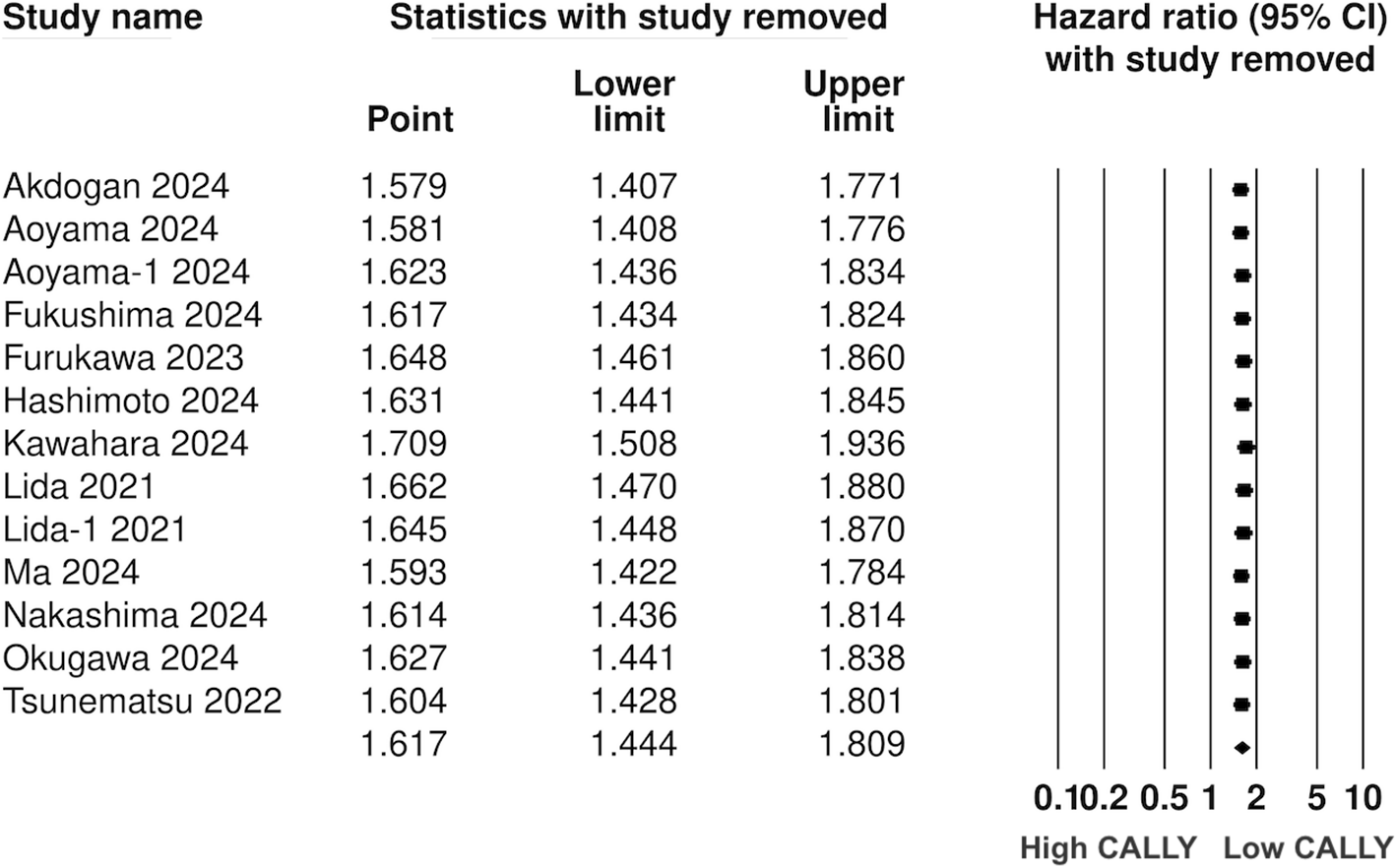
Sensitivity analysis for PFS.

**Figure 7:**
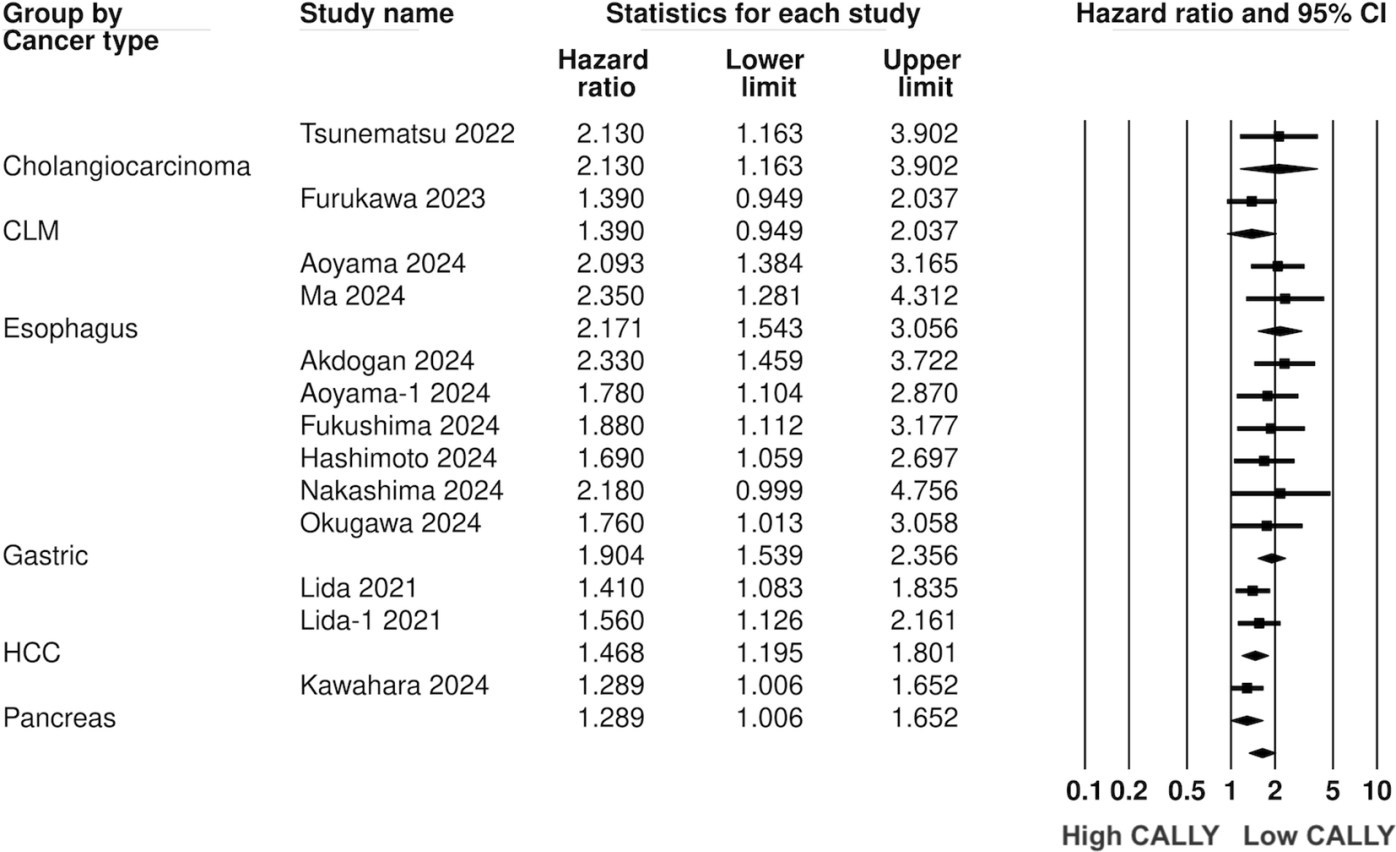
Subgroup analysis for PFS based on different GI malignancies.

Twelve studies (13 cohorts) reported data on PFS. Meta-analysis showed a significantly increased risk of worse PFS with low CALLY (HR: 1.617 95% CI: 1.444, 1.809 I^2^ = 1%) (**Figure 5**). Sensitivity analysis with one study removed sequentially demonstrated robust results (**Figure 6**). Publication bias was not noted on the funnel plot (**Supplementary Figure 2**). Egger’s test was not significant (p=0.63). Subgroup analysis of PFS based on different malignancies is presented in **Figure 7**. Non-significant results were noted for CLM (HR: 1.390 95% CI: 0.949, 2.037) but the effect size remained statistically significant for pancreatic (HR: 1.289 95% CI: 1.006, 1.652), cholangiocarcinoma (HR: 2.13 95% CI: 1.163, 3.902), esophageal (HR: 2.171 95% CI: 1.543, 3.056 I^2^ = 0%), HCC (HR: 1.468 95% CI: 1.195, 1.801 I^2^ = 0%), and gastric cancer (HR: 1.904 95% CI: 1.539, 2.356 I^2^ = 0%). 

The results of the meta-regression analysis are presented in **Table 2**. Sample size, age, male gender, TNM stage III/IV, lymph node metastasis, CALLY cut-off, low CALLY percentage, and follow-up were not found to significantly impact the outcomes.

**Table 2:** Meta-regression analysis.

Moderator	Co-efficient	SE	95% Lower	95% Upper	P-value
OS					
Sample size	0.0000	0.0001	-0.0003	0.0003	0.80
Age	-0.0004	0.0111	-0.0222	0.0214	0.96
Males	-0.0028	0.0046	-0.0118	0.0062	0.53
TNM stage IIII/IV	0.0000	0.0023	-0.0044	0.0044	0.99
Lymph node metastasis	0.0115	0.0089	-0.0059	0.0289	0.19
CALLY cut-off	0.0299	0.0308	-0.0304	0.0902	0.33
Low CALLY %	-0.0058	0.0031	-0.0119	0.0003	0.06
Follow-up	-0.0034	0.0063	-0.0158	0.0089	0.58
PFS					
Sample size	-0.0005	0.0004	-0.0012	0.0002	0.17
Age	-0.0399	0.0244	-0.0839	0.0041	0.07
Males	0.0045	0.0044	-0.0042	0.0132	0.30
TNM stage IIII/IV	0.0076	0.0049	-0.0020	0.0172	0.11
Lymph node metastasis	0.0069	0.0115	-0.0157	0.0295	0.55
CALLY cut-off	-0.0026	0.0809	-0.1612	0.1560	0.97
Low CALLY %	0.0075	0.0132	-0.0184	0.0333	0.57
Follow-up	-0.0045	0.0092	-0.0225	0.0134	0.62

SE, standard error; OS, overall survival; PFS, progression-free survival; TNM, tumor node metastasis.

## Discussion

This study presents the first comprehensive compiled evidence on the prognostic ability of CALLY in patients with GI malignancies. With a detailed database search, we could include 17 recent studies from the literature examining the role of CALLY for various GI cancers. It was seen that patients with low pre-treatment CALLY index were at a significantly higher risk of poor OS and PFS. Importantly, the results were significant for all included GI malignancies except for CLM. The single study on CLM reported a non-significant effect of CALLY on PFS but with an HR of >1 indicating a tendency for worse PFS. The sensitivity analysis added to the robustness of the meta-analysis by demonstrating no change in the significance of the results on the exclusion of any study. The point estimates on sensitivity analysis were in a narrow range for both OS and PFS varying from 1.8-1.9 and 1.5-1.6 respectively; indicating a strong association between CALLY and outcomes. Secondly, the HR for specific malignancies was also significant and in the same range. The pooled analysis showed an approximately two-fold increased risk of poor OS with low CALLY across all GI malignancies. For PFS, a strong association was noted between low CALLY and outcomes in gastric and esophageal cancer but weaker associations were noted for HCC and pancreatic cancer. The latter could be due to the small number of studies available for the meta-analysis.

Previously, only one meta-analysis study has been published on the prognostic value of the CALLY index in cancer patients. Li et al (30) pooled data from six studies on gastric cancer to show that low CALLY was an independent prognostic factor for OS and PFS. Moreover, CALLY has been found valuable in predicting outcomes in non-GI malignancies as well. In metastatic or recurrent head and neck cancer patients treated with pembrolizumab, low CALLY has been associated with significantly shorter OS (8). Mutlucan et al (31) have shown that low CALLY scores were noted in deceased glioblastoma patients as compared to survivors indicating a role of CALLY in assessing prognosis. In a large cohort of non-small cell lung cancer patients, Liu et al (9) have found that CALLY was a significant predictor of survival. Similarly, Tsai et al (32) examined the prognostic ability of CALLY in 279 oral cancer patients and found independent associations between low CALLY and OS as well as PFS. A recent large study (7) from the National Health and Nutritional Examination Surveys from 1999 to 2018 in the USA has shown that low CALLY is a significant predictor of all-cause mortality in various cancer subtypes. The results were found to be robust across different cancers and for both cancer-related and cardiac-related mortality. The results of these studies and the present meta-analysis concur with each other and indicate that CALLY could be an essential tool in the hands of oncologists for determining the prognosis of GI cancer patients.

Other than CALLY, there have been other immune and nutrition based markers used in clinical practice. Of note are prognostic nutritional index (PNI) and controlling nutritional status score (CONUT) which have been widely used. Research shows that PNI can predict OS and PFS in gastric cancer, esophageal cancer, pancreatic cancer as well as CRC (33–36). Another meta-analysis (37) has shown that CONUT is predictive of OS in gastric cancer, esophageal cancer, pancreatic cancer, CRC and HCC. The same study also identified that higher CONUT score was associated with worse PFS in CRC and HCC but not gastric cancer. In comparison, our meta-analysis showed that CALLY was consistently associated with worse OS and PFS in all GI malignancies except for CLM. Till date, there have been no comparative studies assessing the prognostic role of PNI, CONUT and CALLY in the same cohort. Such future studies can provide evidence on the superiority of one score on the other.

One can argue that different cancers have differing prognoses which are further impacted by cancer stage, comorbidities, metastasis, and treatment. Indeed, these factors could be a large source of inter-study heterogeneity. Surprisingly, the inter-study heterogeneity noted in the meta-analysis was low probably due to the consistent prognostic ability of CALLY across all malignancies. Nevertheless, we assessed if any moderators affected the overall pooled analysis by conducting a detailed meta-regression. It was noted that sample size, age, male gender, TNM stage III/IV, lymph node metastasis, CALLY cut-off, low CALLY percentage, and follow-up did not have a significant effect on the outcomes. Of all these moderators, the CALLY cut-off was deemed to be the most important. There was a wide variation in the cut-off of the index ranging from 1 to 6.96. All studies determined the best possible cut-off in their cohorts and still achieved comparable results. Such outcomes are indeed intriguing but consistent with meta-analysis studies (38–41) on other inflammatory biomarkers wherein the best possible cut-off for determining prognosis remains to be identified. We believe that only further research can provide evidence on the best possible cut-off of CALLY to assess prognosis.

The strong association between CALLY and survival outcomes can be attributed to the three components of the index representing inflammatory status (CRP), immune function (lymphocytes), and nutritional status (albumin). All of these have been associated with worse outcomes in malignancies as malnutrition, chronic uncontrolled inflammation, and autoimmune dysfunction can facilitate cancer progression causing cancer cachexia and affecting survival and quality of life (7). Uncontrolled inflammation has been strongly linked with every stage of cancer from inception to metastasis. The plethora of inflammatory mediators and cytokines released during chronic inflammation can aid in the growth of cancer cells by evading the immune system, promoting angiogenesis and metastasis. Moreover, the presence of inflammatory cells can result in oxidative injury by the release of reactive oxygen species and nitrogen intermediates leading to chromosomal injuries, genomic instability, and increased mutation (42, 43). CRP is an acute-phase protein and is considered a marker of inflammation with levels increasing in response to tissue injury, infections, and malignancies. CRP also has an immune regulatory function since high concentrations can inhibit the immune response presented by CD8+ T cells via the FcγRIIb-p38MAPK-ROS signaling pathway (44). Therefore, high levels of CRP reflect an immunosuppressive tumor microenvironment and hence may be associated with worse outcomes in several malignancies (45–47).

Secondly, it is now well-recognized that the patient’s immune system plays a vital role in supplementing drugs for the elimination of cancer cells. The individual’s immune system is therefore an important parameter in assessing the prognosis of cancer. In this context, lymphocytes being the primary immune cell of the body have a major role in immune surveillance (43). T-lymphocytes have a direct action on cancer cells while B lymphocytes release cytokines like interferon-gamma and tumor necrosis factor-alpha which effectively neutralize tumor cells. Lastly, natural killer cells also act against cancer cells bypassing the antigen pathway (42). Lymphocyte counts are used in several indices like SII and SIRI which have been linked with worse outcomes in cancer patients (41, 48). Lastly, albumin levels reflect the individual’s nutritional status and are indicative of malnutrition (49). Malnourished patients have reduced treatment responsiveness, increased chemotherapy toxicity, and poor OS (49).

Our review has a number of strengths and limitations. We have presented the most comprehensive text on the prognostic ability of CALLY in literature. Only adjusted data was pooled to avoid confounding bias. We also conducted subgroup analyses to generate evidence on different GI malignancies and critically evaluated the evidence by a meta-regression analysis. The limitations of the review are the small number of studies for cancers other than gastric, the retrospective nature of data, and our inability to conduct subgroup analyses based on cancer stage, histology, specific treatments, and treatment response. The latter was primarily due to the lack of an adequate number of studies for each cancer subtype and lack of data. Likewise, a longitudinal assessment of the effect of CALLY on outcomes could not be performed as only baseline CALLY was assessed by all studies. There is also a possibility of residual confounding due to unmeasured factors which could have skewed the results. Lastly, a bulk of the data was only from Japan with only one Western study. Hence, we believe that results cannot be generalized at this point.

## Conclusions

CALLY can be used as a biomarker to predict outcomes of patients with GI malignancies. It’s easy availability, low cost, and simplicity coupled with strong and consistent association with survival makes it an apt marker for regular clinical practice. Further research is needed to validate the optimal cut-off of CALLY and to improve the quality of available evidence.

## Data Availability

The original contributions presented in the study are included in the article/**Supplementary Material**. Further inquiries can be directed to the corresponding author.
