# Supplementary figures and images for: Value of CRP, albumin, and lymphocyte index in predicting survival of patients with gastrointestinal malignancies: a systematic review and meta-analysis
Source: Front Oncol. 2025 Jul 16;15:1592794. doi: 10.3389/fonc.2025.1592794 (PMC12307156; doi:10.3389/fonc.2025.1592794)

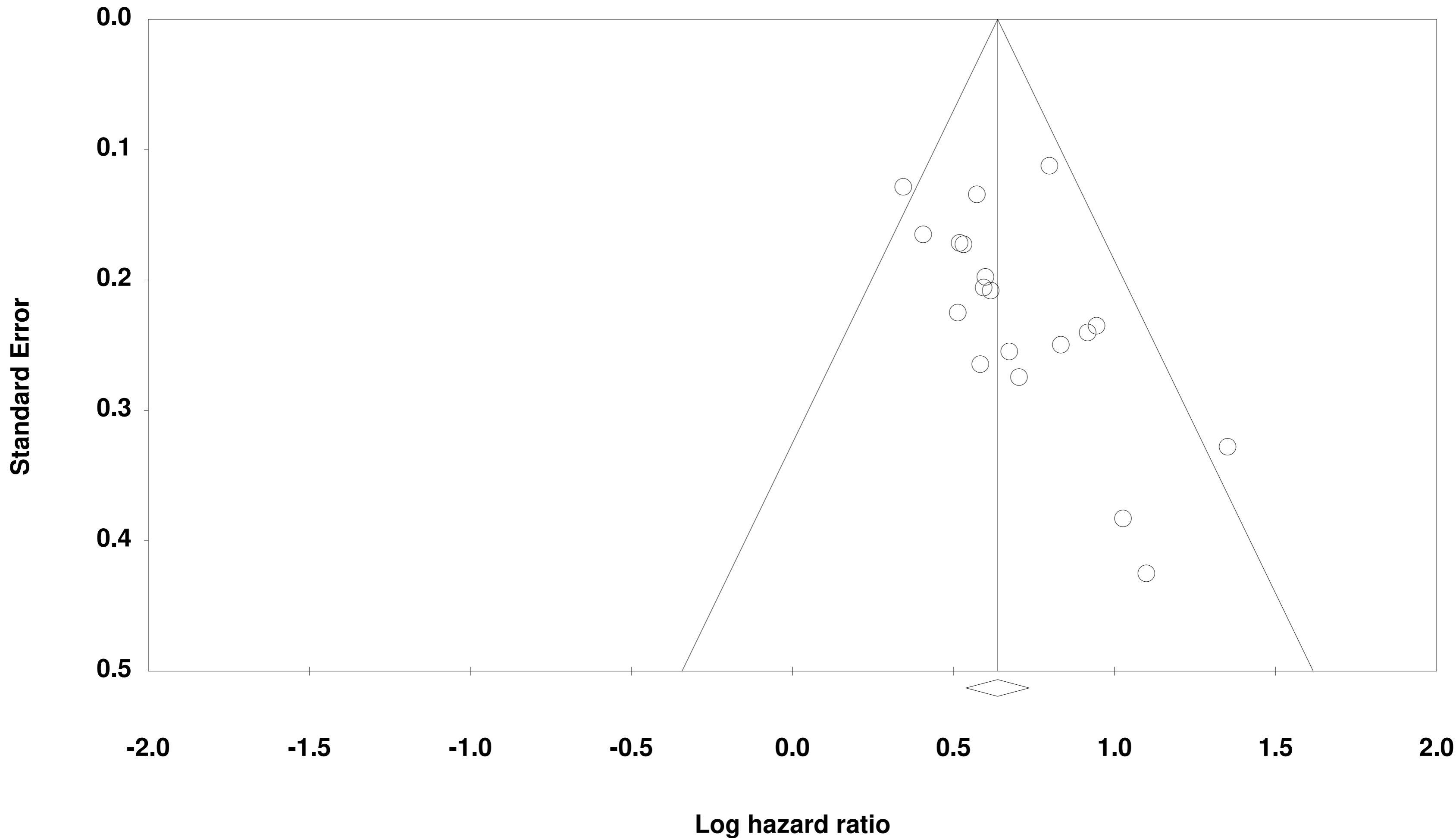

Supplement: Supplementary file 1 [file DataSheet1.pdf]

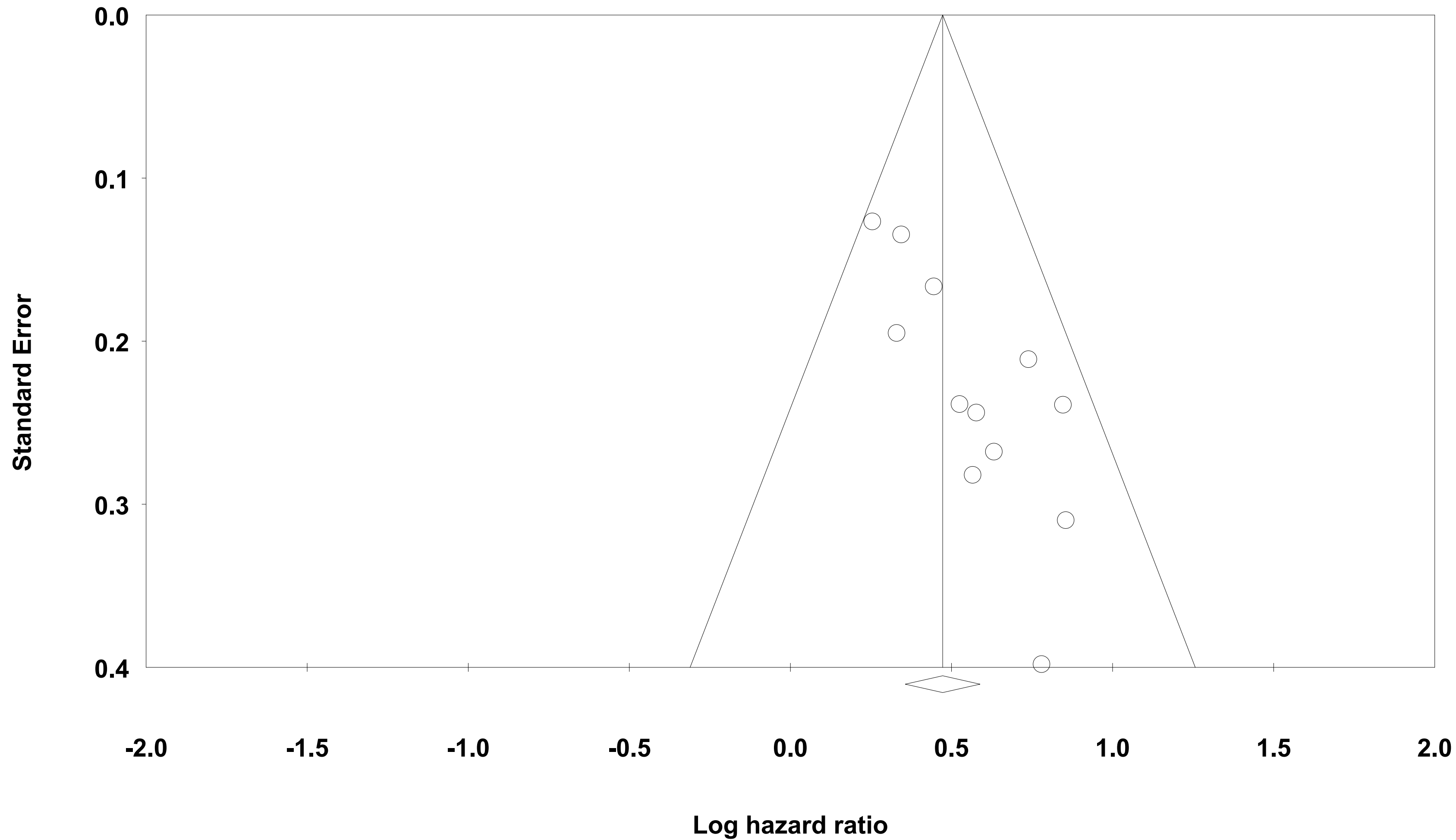

Supplement: Supplementary file 2 [file DataSheet2.pdf]
