# Supplementary material for: Value of CRP, albumin, and lymphocyte index in predicting survival of patients with gastrointestinal malignancies: a systematic review and meta-analysis
Source: Front Oncol. 2025 Jul 16;15:1592794. doi: 10.3389/fonc.2025.1592794 (PMC12307156; doi:10.3389/fonc.2025.1592794)
Supplement: Supplementary file 3 [file Table1.docx]

Supplementary Table 1: Search strategy of all databases

**Embase**

1. 'gastrointestinal cancer'/exp OR 'esophagus cancer'/exp OR 'stomach cancer'/exp OR 'pancreas cancer'/exp OR 'liver cancer'/exp OR 'biliary tract cancer'/exp OR 'colorectal cancer'/exp

2. 'cally index' OR (cally AND ('index'/exp OR index)) OR ('crp albumin lymphocyte' AND index) OR ('c reactive' AND 'protein albumin lymphocyte')

3. #1 AND #2

**PubMed**

1. ((((CALLY index) OR (CRP-albumin-lymphocyte index)) OR (C-reactive protein-albumin-lymphocyte)) AND ((((((((((gastrointestinal) OR (esophageal)) OR (oesophagus)) OR (gastric)) OR (liver)) OR (pancreatic)) OR (hepatocellular)) OR (biliary)) OR (colorectal)) OR (colon))) AND (((cancer) OR (malignancy)) OR (carcinoma))

2. ((CALLY index) OR (CRP-albumin-lymphocyte index)) OR (C-reactive protein-albumin-lymphocyte)) AND ((esophageal Neoplasms[MeSH]) OR (stomach neoplasms[MeSH]) OR (liver neoplasms[MeSH]) OR (pancreatic neoplasms[MeSH]) OR (biliary Tract Neoplasms[MeSH]) OR (colorectal neoplasms[MeSH]))

**Scopus**

(TITLE-ABS-KEY-AUTH (CALLY index) OR (CRP-albumin-lymphocyte index) OR (C-reactive protein-albumin-lymphocyte)) AND (TITLE-ABS-KEY-AUTH (gastrointestinal) OR (esophageal) OR (oesophagus) OR (gastric) OR (liver) OR (pancreatic) OR (hepatocellular) OR (biliary) OR (colorectal) OR (colon)) AND (TITLE-ABS-KEY-AUTH (cancer) OR (malignancy)) OR (carcinoma) OR (neoplasms))

**Web of Science**

((((CALLY index) OR (CRP-albumin-lymphocyte index)) OR (C-reactive protein-albumin-lymphocyte)) AND ((((((((((gastrointestinal) OR (esophageal)) OR (oesophagus)) OR (gastric)) OR (liver)) OR (pancreatic)) OR (hepatocellular)) OR (biliary)) OR (colorectal)) OR (colon))) AND (((cancer) OR (malignancy)) OR (carcinoma))

**Wangfang**

1. 'gastrointestinal cancer' OR 'esophagus cancer' OR 'stomach cancer' OR 'pancreas cancer' OR 'liver cancer' OR 'biliary tract cancer' OR 'colorectal cancer'

2. 'gastrointestinal neoplasms' OR 'esophagus neoplasms' OR 'stomach neoplasms' OR 'pancreatic neoplasm' OR 'liver neoplasms' OR 'biliary tract neoplasms' OR 'colorectal neoplasms'

3. ‘CALLY index’ OR ‘CRP-albumin-lymphocyte index’ OR ‘C-reactive protein-albumin-lymphocyte’

4. #1 OR #2 AND #3
